# Cooking Chicken Breast Reduces Dialyzable Iron Resulting from Digestion of Muscle Proteins

**DOI:** 10.1155/2014/345751

**Published:** 2014-12-28

**Authors:** Aditya S. Gokhale, Raymond R. Mahoney

**Affiliations:** Department of Food Science, University of Massachusetts, Amherst, MA 01003, USA

## Abstract

The purpose of this research was to study the effect of cooking chicken breast on the production of dialyzable iron (an *in vitro* indicator of bioavailable iron) from added ferric iron. Chicken breast muscle was cooked by boiling, baking, sautéing, or deep-frying. Cooked samples were mixed with ferric iron and either extracted with acid or digested with pepsin and pancreatin. Total and ferrous dialyzable iron was measured after extraction or digestion and compared to raw chicken samples. For uncooked samples, dialyzable iron was significantly enhanced after both extraction and digestion. All cooking methods led to markedly reduced levels of dialyzable iron both by extraction and digestion. In most cooked, digested samples dialyzable iron was no greater than the iron-only (no sample) control. Cooked samples showed lower levels of histidine and sulfhydryls but protein digestibility was not reduced, except for the sautéed sample. The results showed that, after cooking, little if any dialyzable iron results from digestion of muscle proteins. Our research indicates that, in cooked chicken, residual acid-extractable components are the most important source of dialyzable iron.

## 1. Introduction

Iron is an essential micronutrient. Most of the iron in the diet is in the nonheme form and is poorly absorbed [1]. Iron absorption can be influenced by a variety of dietary components but it is well accepted that the most effective enhancers of absorption are ascorbic acid and muscle tissue [2, 3]. The effect of muscle has become known as the “meat factor.”

The mechanism of the meat effect remains controversial even after decades of research, largely with* in vitro* systems. Much research suggests that peptides, derived from enzymatic digestion of muscle proteins, chelate iron that would otherwise be insoluble in the upper intestine.* In vitro* studies have indicated that cysteine and histidine residues in peptides could act as iron chelators [4–6]. Peptides could also reduce ferric iron to the more soluble and bioavailable ferrous form through the action of cysteine residues [7]. Both of these mechanisms can lead to increased levels of dialyzable iron. Some studies have suggested that a nonprotein muscle component may be involved [8].

All research involving human subjects (and most of the* in vitro* studies) have used cooked meat or fish as the source material. However, the effect of cooking* per se* has received little attention despite the knowledge that heat causes oxidation of muscle protein sulfhydryls [9] and that sulfhydryls are the structures most often thought to be responsible for the meat effect. We have already shown that heating a homogenized, stirred, muscle slurry in a water bath reduced formation of dialyzable iron [10]. However, those data cannot be used to estimate the effects of cooking intact tissue portions. Cooking is a more complex physical phenomenon than simply heating a slurry since it involves severe temperature gradients, dehydration reactions, browning reactions, and varying alterations in protein structure. Since it is the only practical way of consuming meat, it is important that its effect(s) be evaluated and understood.

Accordingly, we have investigated the effects of cooking on the production of iron binding components during both extraction and digestion. Since cooking methods vary both in temperature and in the method of heating, we have studied the effects of cooking chicken breast muscle by four different procedures, to a common internal temperature of 165°F (74°C), generally accepted as “done.” We used chicken breast as a source of muscle due to its low endogenous iron content. We used dialyzable iron as an indicator of iron species that may be bioavailable. Dialyzable iron correlates well with human bioavailability in systems such as these, where organic acids that chelate iron but do not promote absorption are absent [11].

## 2. Materials and Methods

### 2.1. Materials

Skinless, boneless chicken breast, chilled but not frozen, was obtained from a local supermarket.

Dialysis membranes were from Spectrum Labs, Rancho Dominguez, CA, USA. For digestions to measure dialyzable iron, Spectra/Por 1 membranes with a diameter of 20.4 mm and molecular weight cutoff (MWCO) of 6–8 kDa were used. All membranes were soaked in 5 mM EDTA for 2 hrs and rinsed several times with distilled, deionized water before use.

Pepsin (P 7012), pancreatin (P 1750), bile extract, PIPES buffer, 5,5′ dithiobis (2-nitrobenzoic acid) (DTNB), diethylpyrocarbonate (DEPC), and ferrozine were from Sigma Chemical (St. Louis, MO USA). Iron reference solution containing 1000 ppm ferric iron was from Fisher Chemical, Fairlawn, NJ. All other chemicals were of reagent grade. Distilled, deionized water was used throughout.

### 2.2. Sample Preparation

After trimming to remove visible fat, a single chicken breast was divided into portions of ~100 g of muscle, each 7-8 cm in diameter. A portion of raw chicken was chopped into small pieces of about 4 mm^3^. The chopped sample was then packed in an airtight bag and stored at −40°C. The other portions were cooked to an internal temperature of 165°F (74°C), as measured by a probe, by one of the following four procedures. Boiling: the portion was heated in boiling water. Baking: the portion was heated on a glass baking sheet in an oven set at 365°F and turned over every ten minutes to ensure even heating. Sautéing: a portion was placed in three tablespoons of canola oil heated to a temperature of ≥400°F in a stainless steel pan; the portion was turned over every ten minutes. Deep-frying: a portion was submerged in canola oil heated to 400°F and cooked to the done temperature. A second breast was processed concurrently so that two portions were cooked by each procedure at the same time under the same conditions. All samples were cooled in refrigerator. Fried samples were then patted with paper towels to remove excess oil and then dipped in hexane to remove further surface oil. Once cold, samples were weighed and ground in a spice blender to a size of ~2-3 mm^3^. All samples were individually well mixed and a portion was analyzed for protein content using the Kjeldahl method [12]. The samples were packed in an air-tight bag and stored frozen at −40°C.

### 2.3. Methods

All glassware was soaked in 2 N HCl and then rinsed several times with distilled, deionized water before use.

#### 2.3.1. Digestions

The digestion procedure was based on the method originally described by Miller et al. [13] and included modifications designed to reduce the amount of extraneous iron and control the final pH. The details of the procedure have already been described [14].

A portion of thawed chicken muscle containing 2.0 g protein was homogenized in 80 mL water for 3 minutes, in one-minute bursts. After addition of ferric iron (37.5 *μ*moles/2 g protein) and adjustment to pH 2 at 37°C, the samples were digested at 37°C with pepsin (pH 2) for 2 hours. Adjustment of pH after the pepsin digestion was made with a dialysis bag (6–8 kDa MWCO) containing sufficient NaHCO_3_ to neutralize the titratable acidity [14]. This was followed by digestion with pancreatin/bile/PIPES buffer (pH 6.5) for another 2 hours, as previously described [14]. The final pH after pancreatin digestion was 6.5 ± 0.1.

#### 2.3.2. Controls

An iron-only control was run using the procedures described above but using water in place of the chicken sample.

#### 2.3.3. Extractions

Extracted (nondigested) samples were prepared with chicken muscle and iron using the procedure described under Section 2.3.1 but with omitting the proteolytic enzymes. Accordingly, the sample was extracted at pH 2 for two hours and then partially neutralized with sodium bicarbonate and then extracted for 2 hrs to reach a final pH of 6.5 ± 0.1.

#### 2.3.4. Analysis

After the digestion procedure, the dialyzate and retentate were centrifuged at 1.750 ×g for 10 min to remove insoluble iron. Aliquots of the supernatants containing soluble iron were mixed 1 : 1 with reducing protein precipitant and separately 1 : 1 with nonreducing protein precipitant [14] and then left overnight. The next day the samples were centrifuged again to remove insoluble protein. The final supernatants were analyzed for protein and iron.

Protein was measured by the Biuret method [15] using bovine serum albumin as a standard.

Total and ferrous iron was measured spectrophotometrically with ferrozine as previously described [14] using a standard curve generated using FeC1_3_ (0–5 mg/mL) in the presence of hydroxylamine hydrochloride.

Total sulfhydryls and histidine content were measured spectrophotometrically after protein denaturation as previously described [10].

Nonprotein sulfhydryls were determined by homogenizing muscle samples containing 2.0 g protein with 100 mL cold 0.01 N HCl containing 0.01 mM EDTA for 5 min. The homogenate was centrifuged at 3000 ×g for 10 min and the supernatant was collected. A dialysis tube (6–8 kDa MWCO) containing 20 mL 0.01 N HCl, 0.01 mM EDTA, was placed in the supernatant and dialysis was allowed to continue for 4 hr at 4°C in a shaking Erlenmeyer flask. The dialyzate was diluted fourfold with 0.2 M sodium phosphate, pH 8.0, and sulfhydryls were determined by Ellman's method [16] as described above.

**Figure 1:**
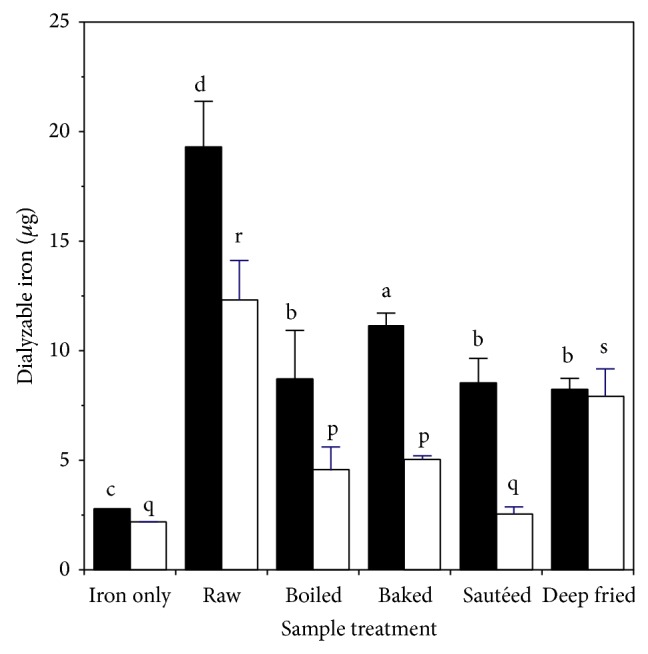
Effect of heating on production of dialyzable iron during extraction of chicken muscle: ■ total dialyzable iron; □ ferrous dialyzable iron. Values are the mean ± standard deviation (*n* = 10). Means in the same group (■ or □) without a common letter differ at *P* < 0.05.

Each experiment was repeated three times unless otherwise stated. Each pepsin digestion was followed by 2 pancreatin digestions of the same sample. Data were analyzed by one-way analysis of variance. Means in Figures 1 through 5 were compared for significance by Tukey's method with a 95% confidence limit. Means in Figure 6 were compared for significance by a *t*-test for two independent samples with a 95% confidence limit.

## 3. Results

The effect of cooking on levels of dialyzable iron after* extraction* is shown in Figure 1. Compared to the iron only control, raw muscle led to about seven times as much dialyzable iron and six times as much dialyzable ferrous iron. About 64% of the total dialyzable iron was ferrous. Cooking led to decreased levels of dialyzable iron for all treatments, in the range 42–57%. Cooking also led to decreased levels of dialyzable ferrous iron for all treatments, in the range 35–79%. The level of dialyzable iron after cooking was greater than the iron-only control for all cooked samples, except for the sautéed sample where dialyzable ferrous iron was not significantly different from the control.

**Figure 2:**
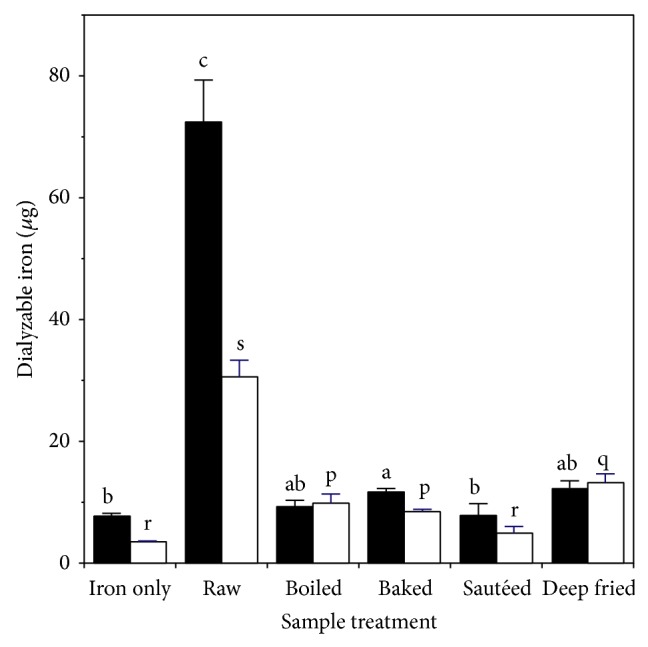
Effect of heating on production of dialyzable iron during digestion of chicken muscle: ■ total dialyzable iron; □ ferrous dialyzable iron. Values are the mean ± standard deviation (*n* = 10). Means in the same group (■ or □) without a common letter differ at *P* < 0.05.

The effect of cooking chicken muscle on levels of dialyzable iron after* digestion* is shown in Figure 2. Compared to the iron only control, raw muscle produced about ten times as much dialyzable iron and nine times as much dialyzable ferrous iron. About 42% of the total dialyzable iron was ferrous. Compared to* extraction* (Figure 1) the* digestion* process led to four times as much dialyzable iron about two and a half times as much dialyzable ferrous iron. Cooking led to decreased levels of both dialyzable iron and dialyzable ferrous iron, regardless of the method employed. For dialyzable iron the decreases ranged from 83% (deep-fried) to 89% (sautéed). For dialyzable ferrous iron the decreases ranged from 58% (deep-fried) to 84% (sautéed). Most of the dialyzable iron remaining after cooking was ferrous. After cooking the levels of dialyzable iron were not significantly different from the iron-only control, except for baked chicken where the values were slightly higher.

**Figure 3:**
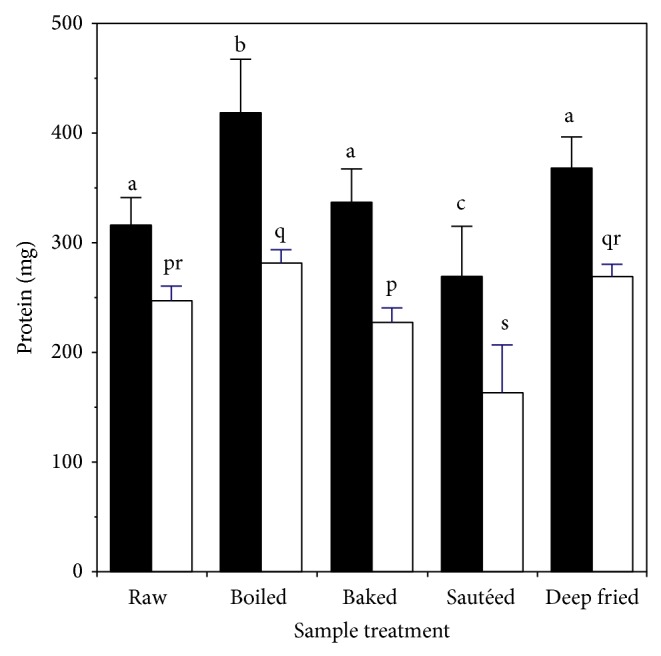
Effect of heating on production of soluble and dialyzable protein during digestion of chicken muscle: ■ soluble protein; □ dialyzable protein. Values are the mean ± standard deviation (*n* = 10). Means without a common letter differ at *P* < 0.05.

The effect of cooking on levels of soluble and dialyzable protein after* digestion* is shown in Figure 3. Compared to the raw chicken, levels of both soluble and dialyzable protein were higher for the boiled samples and lower for the sautéed samples; the values for other samples were unchanged. In cooked samples the proportion of protein that was dialyzable (a measure of the extent of digestion) was in the range 61–73%, which was slightly lower than for the raw sample (78%).

**Figure 4:**
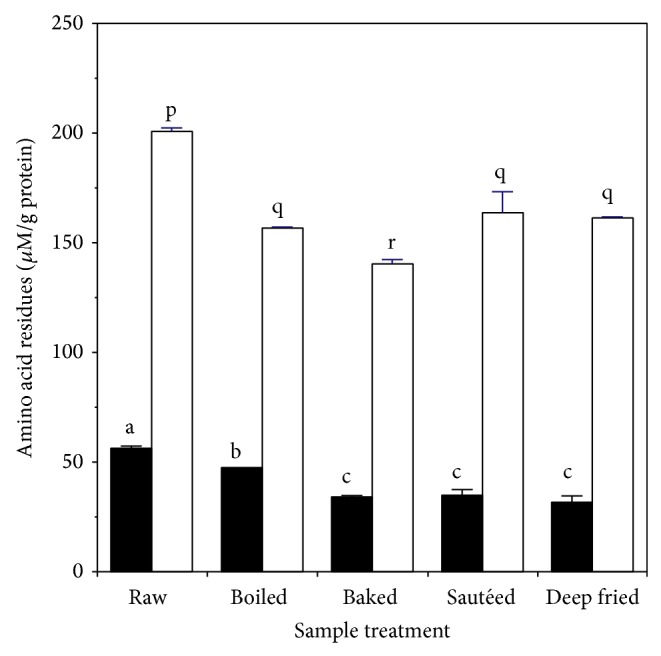
Effect of heating on content of iron-binding amino acid residues in chicken muscle: ■ total sulfhydryl; □ total histidine. Values are the mean ± standard deviation (*n* = 6). Means in the same group (■ or □) without a common letter differ at *P* < 0.05.

The content of total sulfhydryl and histidine residues in the samples is shown in Figure 4. Cooking reduced the levels of total sulfhydryls by about 15% for boiling and by about 40% for all other methods. Cooking reduced the levels of total histidines by 20–30% for all cooking methods.

**Figure 5:**
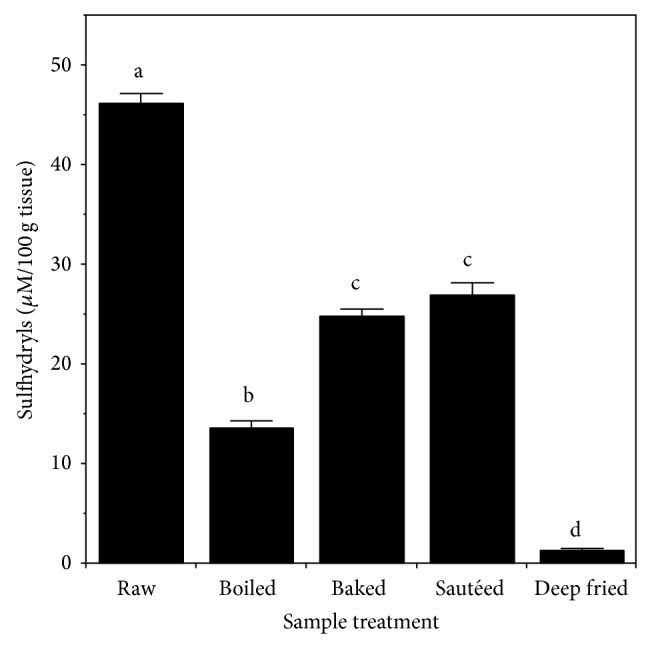
Effect of heating on production of nonprotein sulfhydryls during acid extraction of chicken muscle. Values are the mean ± standard deviation (*n* = 6). Means without a common letter differ at *P* < 0.05.

The effect of cooking on the content of acid-extractable nonprotein sulfhydryls is shown in Figure 5. Cooking caused a marked reduction in sulfhydryls for all samples, especially for deep-fried chicken where the residual level was only 3% of that in raw muscle.

## 4. Discussion

Our studies showed that, with raw muscle, dialyzable iron was produced by both extraction and digestion and thereby confirm earlier reports of two distinct sources of dialyzable iron [17]. The dialyzable iron contained both ferric and ferrous iron species; the ferric is likely to be chelated, while the latter must have been reduced by muscle components.

Dialyzable iron levels after* extraction* were lower for all cooking methods. However, the values were still greater than the control, indicating that cooking had not completely destroyed the muscle components responsible. The nature of these components is uncertain but they must be heat labile and some, at least, must have reducing power. Earlier reports suggest that glutathione [17] or glycosaminoglycans [8] could function as extractable sources of dialyzable iron. Cooking reduced the levels of nonprotein sulfhydryls, which in muscle are known to be principally glutathione [9], and this could well account for the reduced levels of dialyzable ferrous iron after extraction. After extraction there was little difference in dialyzable iron levels between cooking methods, except for the sautéed sample where dialyzable ferrous iron was significantly lower. The sautéed sample was subjected to the highest external temperature because it was in contact with the pan surface that was above 400°F; this extreme heat may explain the lower values observed.

The effect of cooking on levels of dialyzable iron after* digestion* was even more marked than the effect after extraction. The decreases are such that dialyzable iron levels were similar to the iron-only control and indicate that cooking had largely destroyed the enhancing effect of chicken muscle proteins. There was no significant difference between remaining levels of dialyzable iron and remaining levels of dialyzable ferrous iron in the same cooked samples, indicating that essentially all the dialyzable iron was ferrous. This indicated that cooking affected components that chelate ferric iron as well as those that reduce ferric iron and that the effect on the former was especially marked.

Cooking can destroy heat-labile amino acid residues. We observed decreased levels of histidine after cooking for all samples. Histidine residues in peptides from muscle have been shown to contribute to iron binding [4] so it is likely that the destruction of histidines contributed, in part at least, to the reduction in levels of dialyzable ferric iron.

The decreased levels of ferrous iron in digested cooked samples could be due to the reduced levels of total sulfhydryls, which are principally cysteine residues of muscle proteins and which are known to be heat labile [9]. The loss of sulfhydryls was least for the boiled sample, which can be expected since it was exposed to the lowest temperature.

The percentage decreases in histidine and sulfhydryl residues in the cooked samples were less than those observed for homogenized chicken muscle slurry that was heated to the same internal temperature of 165°F [10], indicating that the amino acid residues in the intact tissue were less heat labile. On that basis it might be expected that dialyzable iron levels from intact cooked chicken breast would be greater than from homogenized chicken. The results show the opposite, which implies that the decreased levels of dialyzable iron cannot be accounted for simply by destruction of those amino acid residues.

**Figure 6:**
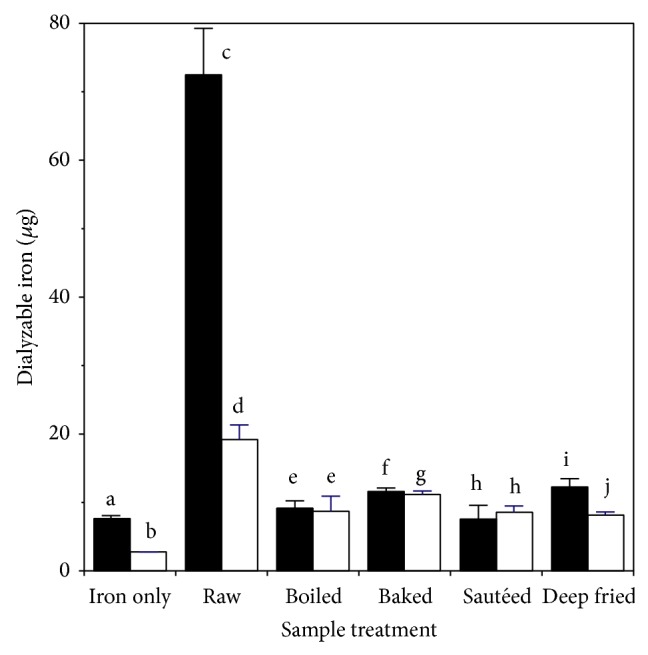
Effect of heating on production of dialyzable iron during digestion and extraction of chicken muscle: ■ digestion; □ extraction. Values are the mean ± standard deviation (*n* = 10). Means in the same treatment group (■ and □) without a common letter differ at *P* < 0.05.

By comparing the levels of dialyzable iron after* digestion* with the levels after* extraction*, as shown in Figure 6, it is possible to* estimate by difference* the dialyzable iron resulting from the proteolytic part of the digestion process. It is clear that in the cooked samples the levels of dialyzable iron after* extraction* and* digestion* were essentially the same (the means differ by less than one *μ*g iron) except for the deep fried sample; in that sample there was a difference of 3 *μ*g but it was still less than the difference in the iron-only control (5 *μ*g). This comparison indicates that whereas proteolytic digestion of the raw sample led to a large increase in dialyzable iron there was little or no effect of digestion in the cooked samples; that is, the effect was mostly negated by cooking.

Levels of dialyzable iron after cooking were very similar for all cooking treatments despite the differences in temperature to which the samples were exposed during cooking. This indicates that the internal temperature reached, which was the same for all samples, may be the more important factor in determining the eventual levels of dialyzable iron.

The effect of cooking on levels of dialyzable protein varied according to the treatment. Decreased digestion would lead to decreased levels of peptides and since these may be responsible for iron chelation they could contribute to lower dialyzable iron. However, reduced dialyzable protein levels were observed only for the sautéed sample, so it seems unlikely that impaired protein digestion was an important factor for the other samples.

Our findings for the effect of cooking are in clear contrast to other studies that reported the effect of cooking on dialyzable iron. Kapsokefalou and Miller [18] reported that cooking beef by broiling and microwaving had no effect on dialyzable ferrous iron levels after digestion, whereas we found a marked reduction for all treatments. They found that broiling caused a ~25% reduction in total dialyzable iron, to levels that were still significantly higher than the control. We found that all cooking methods reduced dialyzable iron to levels that were not different from the control, except for the baked sample that was slightly higher.

Sørensen et al. [19] determined the effect of heating pork meat, at temperatures in the range 60–120°C, on the production of dialyzable ferrous iron from exogenous ferric chloride. They found that increasing heat treatment led to* increased* dialyzable ferrous iron after pepsin digestion and suggested that this might be due to increased accessibility to proteolysis. However, after a pepsin-pancreatin digestion they found only very low levels of dialyzable iron in raw muscle and no effect of heat treatment. In contrast, we found high levels of dialyzable iron in raw muscle and a marked reduction after cooking. Sørensen et al. [19] also reported that heating pork at 70°C and 90°C caused an increase in thiol groups, whereas we found that all cooking methods caused a decrease in thiols.

## 5. Conclusions

The dialyzable iron obtained using cooked chicken muscle is derived mostly from dialyzable source molecules that are extractable in acid, but it is markedly reduced compared to raw muscle. Cooking negates the ability of digested muscle proteins to produce iron-binding peptides that contribute to the formation of dialyzable iron, though the mechanism of the heating effect is uncertain. Consequently, in systems where dialyzable iron is a reasonable proxy for iron bioavailability, cooking chicken muscle would markedly reduce the effect of “the meat factor,” mostly through its negative effect on the muscle proteins.
